# Poly(vinyl alcohol) freeze casts with nano-additives as potential thermal insulators

**DOI:** 10.1038/s41598-022-27324-2

**Published:** 2023-01-19

**Authors:** C. Hübner, M. Vadalà, K. Voges, D. C. Lupascu

**Affiliations:** Institute for Materials Science and Center for Nanointegration Duisburg-Essen (CENIDE), University of Duisburg-Essen, 45141 Essen, Germany

**Keywords:** Chemistry, Materials science

## Abstract

Freeze-casting consists of freezing a liquid suspension (aqueous or other), followed by sublimation of the solidified state to the gas state under reduced pressure, and subsequent sintering of the remaining scaffold to consolidate and densify the struts and walls. The structure is very porous with the pores being a replica of the solvent crystals. The technique is rather versatile and the use of a liquid solvent (water most of the time) as a pore forming agent is a strong asset. Freeze-casting has also been developed as a near net shape forming route yielding dense ceramics. In this work we report on porous composite materials synthesized via the ice templating method. Poly(vinyl alcohol) (PVA) is used as matrix and nano-silica (SiO_2_), nanoclay (NC) and microfibrillated cellulose (MFC) are used as fillers to improve the mechanical stability of the PVA scaffold. We show our results on the porosity and mechanical stability and consider these porous nanocomposites as potential insulation materials with low thermal conductivity and superior mechanical properties.

## Introduction

Freeze casting, also known as ice templating, is a technique to introduce selective porosity into different kinds of materials^1–4^. Porous ceramics^5–9^, porous metals^10–12^, polymers^13–15^, and organic–inorganic composites^16–21^, have been studied in the last 20 years.

The ice templating process is divided into three main steps. First the particles or polymers are dispersed or dissolved in a solvent. Then the precursor dispersion/solution is exposed to a temperature gradient by placing a mould on a cold finger which is dipped into a freezing agent, generally liquid nitrogen^22^. During this process the ice forms elongated crystals along the freezing direction from the cold finger to the top of the dispersion/solution due to constitutional supercooling at the ice-water-interface. Finally, the frozen solvent is removed via freeze drying. The most important advantage of materials which are synthesized by ice templating is the improvement of the mechanical stability compared to materials with isotropic porosity, which can be increased by up to 400%^14^.

Porous polymeric structures formed by freeze-casting are used for different applications. The open pore structure of an ice templated poly(vinyl alcohol) PVA composite or gelatin^23^ can be used for the targeted release of pharmaceutical ingredients or for skin regeneration materials^24^. In one of our previous works, we showed that crosslinked PVA freeze-casts are potential insulation materials^25^. They have a low thermal conductivity in the range of commonly used insulation materials but show significantly better mechanical stability compared to other foam-like insulation materials.

To further improve the properties of the porous materials, different inorganic or organic fillers can be added during synthesis. Nanoparticles like SiO_2_^6^ or carbon black^26^, cellulose nanofibers^27,28^, or silicate minerals^29,30^ can be used. The insertion of SiO_2_^31^ leads to improved mechanical stability of PVA scaffolds. Sun et al.^31^ synthesized PVA aerogel/silica nanocomposites by growing a silica coating onto PVA scaffolds via freeze-casting and were able to increase the compressive strength from 1.8 to 6.0 MPa. The introduction of cellulose nanofibers into PVA scaffolds leads to improved mechanical stability, too^27^. Hostler et al.^18^ reported of a composite material made of PVA and nano-clay via freeze-casting. The material has a very low thermal conductivity of 0.030 W m^−1^ K^−1^ perpendicularly to the freezing direction.

In this work we report on porous composite materials synthesized via the ice templating method. For this purpose, PVA is used as matrix and nano-silica (SiO_2_), nanoclay (NC) and microfibrillated cellulose (MFC) are used as filler materials to improve the mechanical stability of the PVA scaffold. We evaluate these porous nanocomposites as potential insulation materials with low thermal conductivity and superior mechanical properties.

## Materials and methods

Poly(vinyl alcohol) with an average molecular weight of 13.000–23.000 g mol^−1^ (98% hydrolyzed, Sigma Aldrich) was used. Nano-silica AEROSIL®MOX 80 was purchased from Evonik (primary particle size ~ 30 nm). As silicate mineral, hydrophilic bentonite from Sigma Aldrich was used. The micro fibrillated cellulose fibers were from Weidmann Fiber Technology AG (3.21 wt.% in water). All experiments were performed using deionized water.

The synthesis of porous PVA scaffolds without filler was performed equal to our previous work^25^. PVA stock solutions with different PVA amounts in water (5 wt.%, 7.5 wt.% and 10 wt.%) were poured into a PTFE mould, which was placed on a brass cylinder dipped into liquid nitrogen. Finally, the freeze cast samples were freeze dried under vacuum.

PVA-stock solutions were prepared with different PVA amounts in water (5 wt.%, 7.5 wt.% and 10 wt.%) analogous to what we described above. The PVA pellets were dissolved in deionized water under stirring at 90 °C for several hours. For the synthesis of the composite materials, the stock solutions were cooled to room temperature and then mixed with the filler materials. In the case of nano-silica, the SiO_2_ particles were weighed in different weight ratios with respect to water (5 wt.% and 10 wt.%) in a round bottom flask, and the PVA stock solution was added. After homogenization with an ultra-sonic tip (Bandelin Sonoplus HD 2200, 16 kHz) for 15 min, the SiO_2_/PVA/water dispersions were poured into a PTFE mould, placed on a brass cylinder that was dipped into liquid nitrogen. At the bottom of the mould the temperature was kept constant at around  − 175 ± 5 °C. Under these conditions, the ice front moves upwards with an average velocity of about 30 µm/s. The completely frozen samples were placed into vacuum (~ 0.1 mbar) for at least 72 h to let the ice completely sublimate.

The micro-fibrillated cellulose (MFC) was purchased as 3.21 wt.% dispersion in water. The amount of water in this dispersion must be taken into consideration during weighing. The cellulose fibers were used with 0.5 wt.% and 1 wt.% with respect to the total amount of water in the MFC/PVA/water dispersion. All other synthesis steps were equal to the described preparation of SiO_2_/PVA composites.

If hydrophilic bentonite (nanoclay, shortly NC) is used as filler, the synthesis route must be changed, because no stable dispersions of NC/PVA/water can be prepared by mixing the pure silicate minerals with the PVA stock solutions. Stable NC/PVA/water dispersions were obtained by the preparation of a 5 wt.% stock solution of NC and the following mixture with the PVA/water stock solutions. Silicate minerals were used with 2.5 wt.% and 5 wt.% with respect to water in the NC/PVA/water dispersion.

The thermal conductivity of the composites was measured by light flash analysis (LFA 467 Hyperflash, Netzsch). The morphology of the materials was analyzed by scanning electron microscopy (ESEM, FEI Quanta 400F). The pore size distributions were determined by mercury porosimetry (Pascal 440, Thermo Finnigan). The compressive strength was determined uniaxially as stress–strain curves. For the mechanical analysis, the samples were cut into cubes with an edge length of 15 mm and the faces were polished plane parallel.

## Results and discussion

### Morphology

**Figure 1:**
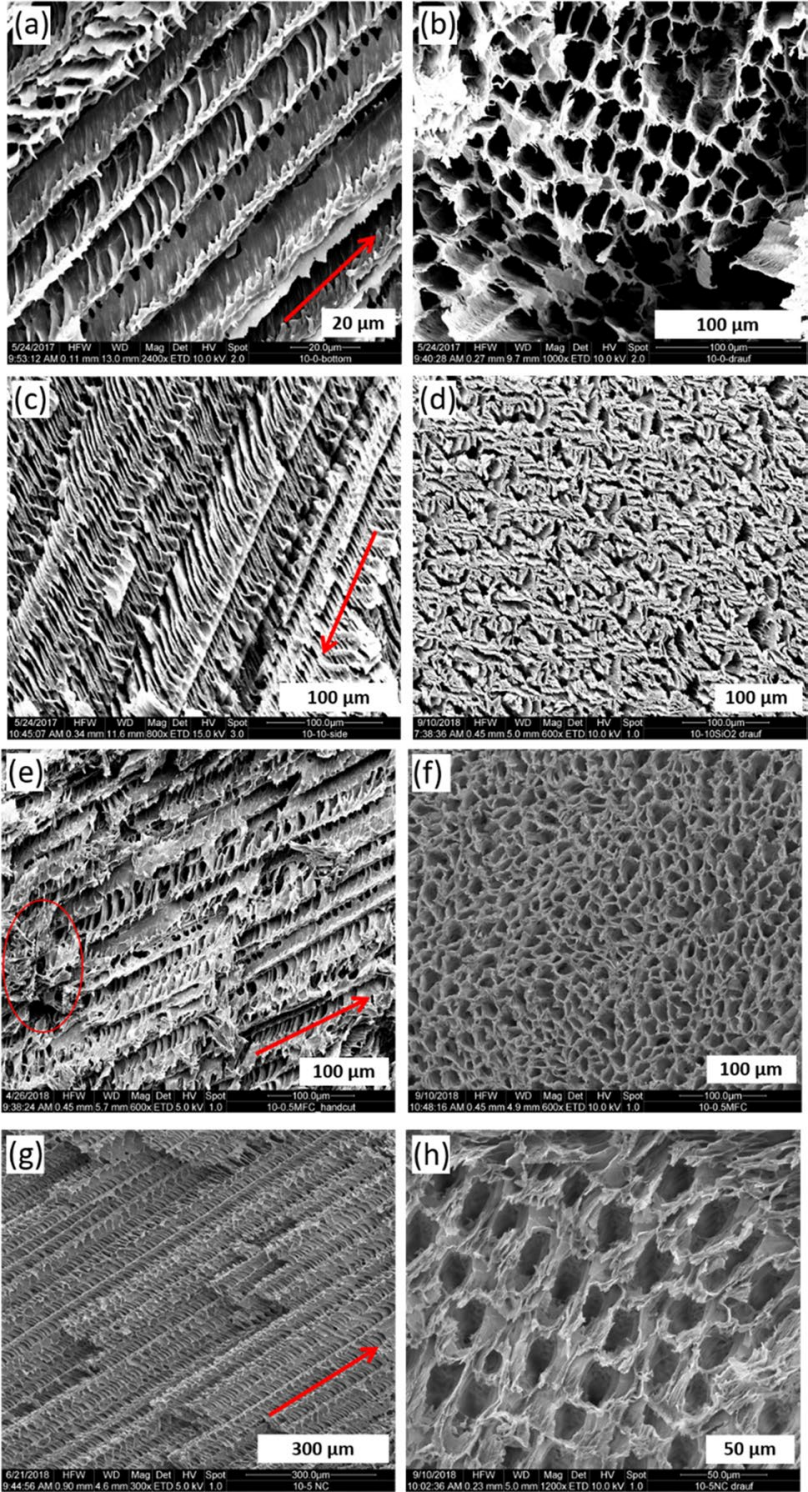
SEM images of the material morphologies: parallel and cross cuts. All materials were prepared using 10 wt.% PVA. Red arrows represent the freezing direction. (**a**) and (**b**) pure PVA, (**c**) and (**d**) SiO_2_/PVA, (**e**) and (**f**) MFC/PVA, (**g**) and (**h**) NC/PVA.

A representative image of pure PVA scaffolds (10 wt.% PVA) is shown in Fig. 1a. The polymer forms a porous structure with parallel pore channels, which are generated by the unidirectional freezing process. The pore channels have diameters between 15 and 40 µm. The walls of the channels show an undulated structure. This morphology is caused by the dendritic growth of the ice crystals during the freezing process. The cross section of the material shows that the pore channels are arranged in a honeycomb like structure (Fig. 1b). A decrease of the PVA amount leads to materials with decreased density and bigger pores.

When nano-silica is added to the PVA/water dispersion, the morphology of the freeze cast composite materials changes. In this case, the growth of unidirectional pore channels is observed as well, but there are additional parallel structures at an angle of approximately 60° with respect to the freezing direction (see Fig. 1c).

These angled substructures appear in periodic recurrences of 5–10 µm. The morphology of the composite overall looks like a fishbone. The SiO_2_ nanoparticles influence the growth kinetics of the ice during the freezing process towards an increased dendritic character of the growth. The cross sections show, that the SiO_2_/PVA composite has no longer the typical honeycomb like structure (Fig. 1d).

The inclusion of micro fibrillated cellulose fibers shows no significant changes of the morphology of the PVA scaffolds. The PVA forms unidirectional and parallel pore channels, and the cross section shows the honeycomb structure as well (see Fig. 1e and f). This structure is sometimes interrupted by clew-like accumulations of agglomerated cellulose fibers.

The morphology of the composites consisting of PVA, and NC show the formation of many parallel pore channels in the freezing direction. Additionally, transverse structures, like the composites with SiO_2_, can be observed, but the cross section of the material is more honeycomb like than diffuse (see Fig. 1g and h).

### Porosity

The pore sizes and pore size distributions of the pure PVA scaffolds and the influence of the different filling materials on the porosity were investigated by means of mercury intrusion porosimetry. The mean pore size of PVA scaffolds is in the range between 8 and 20 µm. This is in good agreement with the SEM results. When the PVA amount in the material decreases, the pore sizes increase significantly, and most of the pores show diameters between 20 and 100 µm. This result is in good agreement with our expectations that the pore sizes within the materials depend on the PVA amount and increase when the solid content decreases.

As expected, the incorporation of SiO_2_ nanoparticles into the PVA matrix leads to a decrease of the total pore volume within the material. This effect is more pronounced the more nanoparticles are used and can be explained by the increase of the total solid content in the system. The sizes of the pore channels decrease with the amount of SiO_2_. Furthermore, pores with diameters lower than 1 µm are observed. These small pores were observed only in the composite material and not in the pure PVA scaffolds.

In both cases, the pore size distributions of the composites are narrower than in the SiO_2_/PVA composites and no small pores under 1 µm are observed. The fact, that no small pores occur with MFC and NC as filler materials, can probably be explained by the structure of the filling materials. The formation of voids between particles or particle agglomerates of the straight cellulose fibers and the silicate mineral nanoplates is not that distinctive as in the case of the silica agglomerates.

### Thermal conductivity

For the estimation of the thermal conductivity of the composites, the thermal diffusivities $\alpha$ were measured via Laser Flash Analysis (LFA) experiments. Using estimations of the specific heat capacity (*c*_*p*_) and the density of the materials, the thermal conductivity $\lambda$ of the composites can be calculated.

1 $$\lambda = \alpha \cdot c_{p} \cdot \rho$$

**Figure 2:**
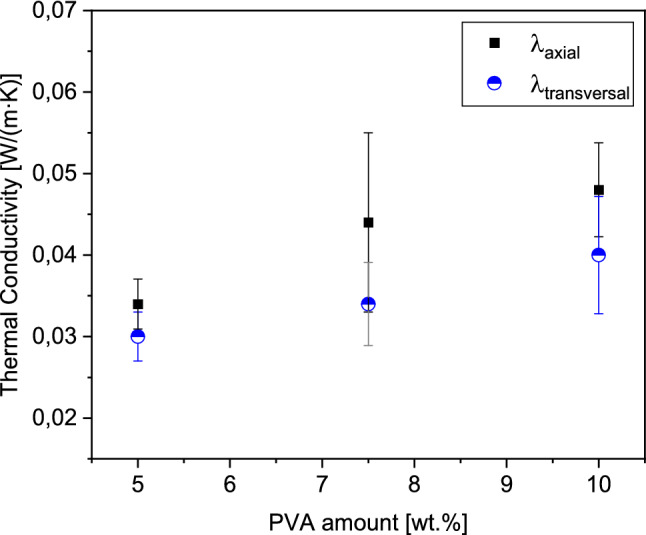
Calculated thermal conductivities of the pure PVA scaffolds in dependency of the PVA amount.

The thermal conductivity of the pure PVA scaffolds increases with the PVA amount which is used to synthesize the materials (see Fig. 2). Furthermore, the thermal conductivities of the materials are higher in the axial direction than in the transverse direction. The heat flow along the pore channels is more continuous and not hindered, as it is perpendicular to the pore channels. In the latter direction, the heat flow is reduced by trespassing the air-filled pores multiple times, which results in a lower thermal conductivity.

The results are in good agreement with the estimation of the porosity shown before. The less PVA is used to synthesize the material, the higher is the porosity within the system, and thus the lower is the thermal conductivity.

**Figure 3:**
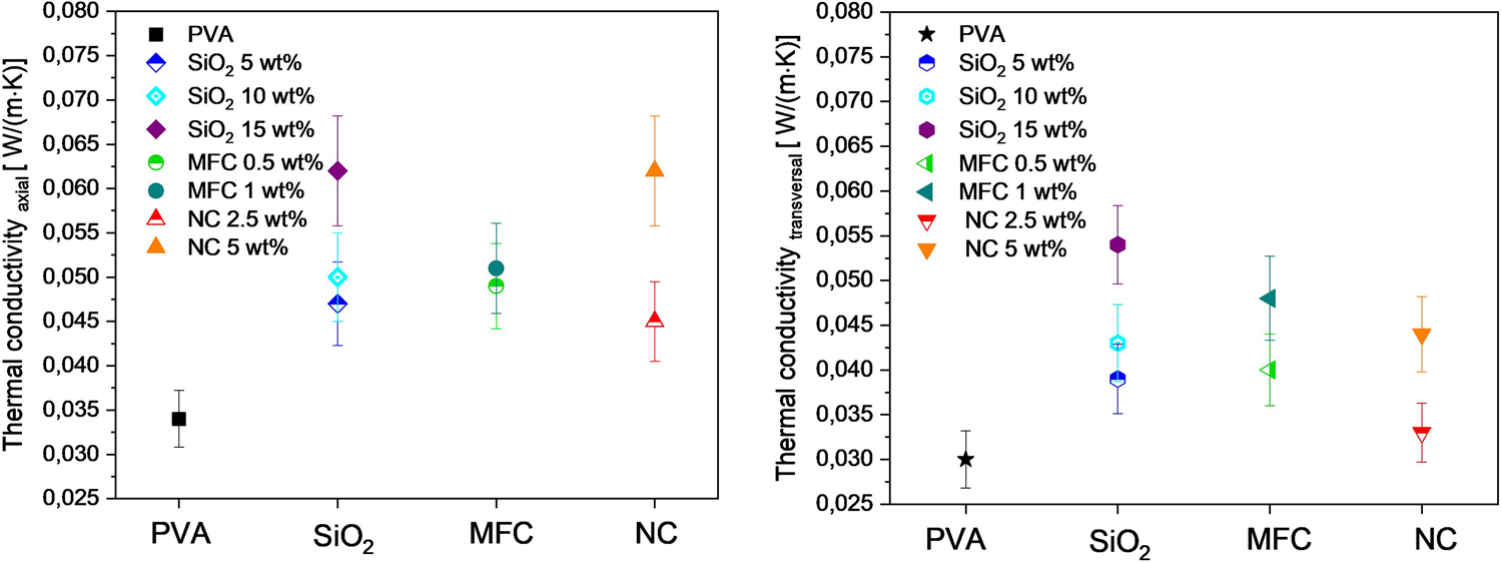
Thermal conductivities (calculated from the measured thermal diffusivity) in axial (left) and transversal (right) direction of the composites with 5 wt.% PVA. Pure PVA scaffolds (square and star black symbols), PVA/SiO_2_ composites (rhombus and hexagon blue, cyan and purple symbols), PVA/MFC composites (dark green and green circle and pointing left triangles) and PVA/NC composites (red and orange pointing un and pointing down triangles).

The thermal conductivities of composite materials are graphically summarized in Fig. 3. On the left side, the thermal conductivity in axial direction and on the right side the thermal conductivity in transverse direction are shown for the composites which contain 5 wt.% PVA. The incorporation of the three fillers (colored symbols) in general leads to an increase of the thermal conductivity in comparison with the pure PVA scaffolds (black symbols). Furthermore, the increase is stronger when the amount of filler material is increased. Over-all, the thermal conductivity of the materials is higher in the axial direction than in the transverse direction.

### Mechanical properties

**Figure 4:**
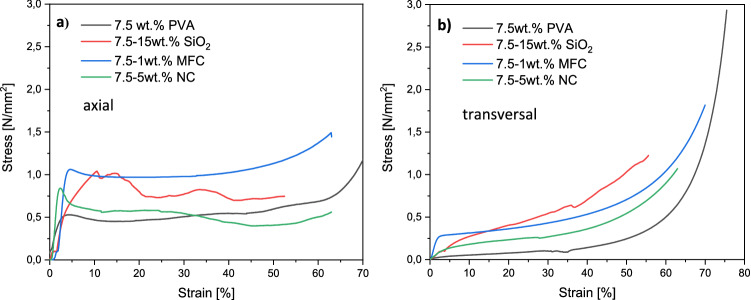
Stress–strain-curves in axial (**a**) and transversal (**b**) directions for the composites with 7.5 wt.% PVA. Pure PVA scaffolds (black), PVA/SiO_2_ composites (red), PVA/MFC composites (blue), and PVA/NC composites (green).

To investigate the mechanical strength of the materials, uniaxial compression tests in axial and transverse directions were performed (see Fig. 4).

The axial stress–strain-curves of the pure PVA samples show a linear increase at the beginning, reflecting the elastic range. After a maximum is reached, the curve falls off yielding an irreversible structural damage. At higher strains, the compressive stress finally increases again due to the compaction of the material by closing the pores (Fig. 4a, black curve). The increase of the transverse strain curve is flatter at the beginning and the stress values are significantly lower (Fig. 4b, black curve). The curve does not go through a maximum but reaches a plateau with a small continuous increase in strain. Finally, the stress again increases more by the compression of the material. This difference between the two directions of stress well reflects the anisotropy of the structure.

The addition of SiO_2_ particles significantly changes the stress–strain curves. The increase of the axial strain at the beginning is slightly curved at high particle contents of 15wt.%. This indicates a certain amount of permanent deformation before the maximum stress is reached. Furthermore, the curve shape after reaching the maximum is significantly irregular for high particle content (Fig. 4a, red curve). The reason for this could be the failure of smaller partitions of the samples leading to small strain drops. Thus, the silica/polymer samples show a certain brittle fracture behavior. For transversal strain, the stress–strain-curves of the silica freeze casts show a similar trend as the pure PVA samples. For great deformations, the stresses again increase due to material compaction (Fig. 4b, red curve).

The stress–strain curves of the freeze casts with MFCs behave very similar to those of the pure PVA—they also proceed very smoothly and without any irregularities. This is certainly due to the flexibility of the fibers, which does not lead to the failure of individual zones as with the silica samples, but only to plastic deformation (Fig. 4a and b, blue curve). The samples with NCs behave more like the SiO_2_ freeze casts and the curves are also rather irregular (Fig. 4a and b, green curve).

As reference, the effect of talc on the crystallization behavior of PVA and its regulation mechanism on the crystallization properties of PVA films were investigated in^32^. The authors could prove that, when adding talc nanoparticles as nucleation agent, the crystallinity of PVA increases. In^33^ PVA fibers were modified to be able to adsorb heavy metals. Considering this literature, we certainly assume that, if appropriately modified, our PVA matrices could be utilized also as filters.

### Failure mechanisms

**Figure 5:**
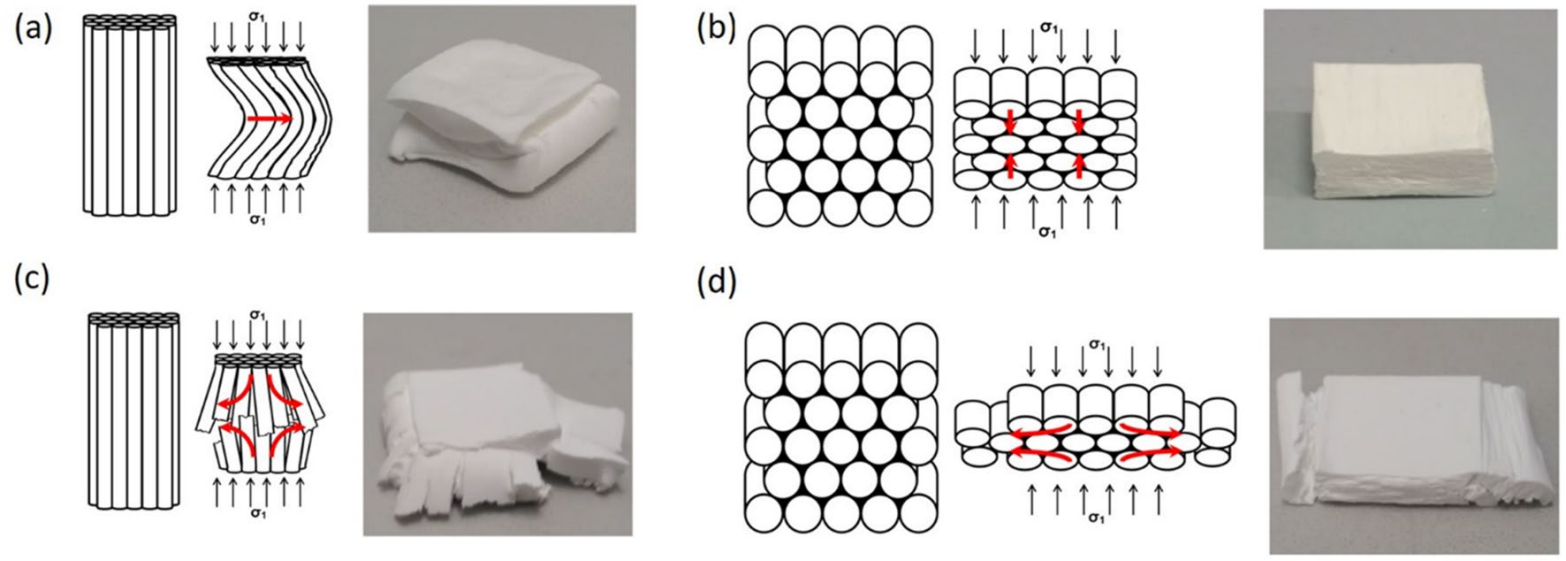
Schematical description of the failure behavior: (**a**) Pure PVA and PVA/MFC (axial) (**b**) Pure PVA and PVA/MFC (transversal), (**c**) PVA/SiO_2_ and PVA/NC (axial), and (**d**) PVA/SiO_2_ and PVA/NC (transversal).

The application of axial strain to the particle-free freeze casts leads to a compression of the pores and to laterally buckled structures without recognizable cracks (Fig. 5a). The compression is irreversible, indicating that the material is more plastic and shows little elasticity. In the case of transversely applied stress, the sample is compressed in the stress direction. Obviously, the pore channels are only compressed in this case—an elongation in the lateral direction does not take place. Even with transverse loading, the deformation is not reversible (Fig. 5b).

MFC samples behave almost identically under uniaxial compressive stress and show similar deformations.

If the particle-containing freeze casts are loaded axially, a significantly different damage pattern is observed (Fig. 5c). They behave increasingly brittle with increasing particle content and final disintegration of the structures. The material splits into larger bundles along the stress direction and, the pore channels finally break perpendicular to it. The higher the particle/polymer ratio, the more individual bundles are formed and the smaller they become. This indicates that the buckling strength decreases perpendicular to the pressure load with increasing particle content, which is understandable as less and less polymer is available for the cohesion of the particles. Thus, larger unbound particle agglomerates serve as weak points in the pore walls, where material failure occurs.

Transverse loading of these samples also results in compression in the stress direction, but in contrast to the pure PVA an additional displacement of material in the lateral direction occurs (Fig. 5d). Entire bundles are pushed out of the side of the structures. The bundles become thinner with larger particle/polymer ratio. The freeze casts with NC shows a similar behavior.

### Compressive strength

In all freeze casts of this study, we observed a linear behavior for compressive strength, meaning that both in axial and transverse directions compressive strength strongly increases with the PVA content. The compressive strength in axial direction is significantly higher than in the transversal direction. The addition of silica particles increases the strength compared to pure PVA. The particles serve as a framework, which makes the buckling of the pore walls more difficult and thus leads to an increase in strength. The maximum values of the failure stress are present under axial load of the 15 wt.% SiO_2_ samples, with nearly 1.9 N/mm^2^.

Selected values for the compressive strength of all materials are summarized in Table 1.

**Table 1:** Values of the compressive strengths in both directions (axial and transversal) with different amounts of PVA and the maximal degree of filling for all filler materials.

Sample	Axial strength	Transversal strength
	[N/mm^2^]	[N/mm^2^]
5–0	0.07 ± 0.01	0.04 ± 0.01
7.5–0	0.47 ± 0.09	0.04 ± 0.02
10–0	0.93 ± 0.06	0.12 ± 0.01
5–15 SiO_2_	0.74 ± 0.27	0.18 ± 0.04
7.5–15 SiO_2_	0.84 ± 0.27	0.22 ± 0.02
10–15 SiO_2_	1.88 ± 0.22	0.37 ± 0.20
5–1 MFC	0.36 ± 0.07	0.11 ± 0.01
7.5–1 MFC	0.99 ± 0.09	0.27 ± 0.01
10–0.5 MFC	1.32 ± 0.01	0.33 ± 0.01
5–5 NC	0.49 ± 0.01	0.04 ± 0.01
7.5–5 NC	0.83 ± 0.02	0.13 ± 0.01
10–5 NC	0.89 ± 0.03	0.31 ± 0.03

By adding MFC, the compressive strength in the axial and transverse direction can be increased, as well. The fibers primarily increase the strength of the PVA^34^, which means that deformation of the material and buckling of the pore walls seems to occur only at higher stresses. While the addition of 0.5 wt.% of fibers has a strong influence on the strength, a further increase to 1 wt.% provides only minor improvements. Thus, the required amount of fiber can be kept relatively low.

The addition of NC only leads to a slight improvement in mechanical properties. Samples with 2.5 wt.% NC exhibit similarly low strength as pure PVA freeze casts and sometimes even show a decrease in compressive strength in axial direction. When 5 wt.% NC are used, a recognizable increase in the compressive strength can be observed, although here the values for the 10–5 NC are below the 10–0 sample. Maybe these samples already had cracks inside, which were not visible from the outside. A reason for the low impact on of NCs on the mechanical strength could be, that the small number of two-dimensional platelets can hardly form a supporting particle framework. In addition, flat clay plates offer the possibility of sliding over each other, which only requires low shear forces when no polymer is in between.

The results of the uniaxial compression tests are comparable to the anisotropic behavior of wood. Wood is also an anisotropic material with large differences in mechanical properties in the axial and transverse directions, whereby the compressive strength along the fiber direction (axial) can be 2- to 20-times higher. A comparison of the stress–strain curves of the freeze casts and of wood shows that the tangential curve of the wood has a related shape to the transversely loaded freeze cast specimens. The axial curve of wood is similar to the curve of the particle loaded PVA samples, which also show an irregular and ridged profile after reaching the first maximum. The freeze-casts without particles have a smoother shape, suggesting that their failure mechanism is more plastic and less brittle than the one of the cell walls of wood. The mechanical behavior of wood under pressure can be simplified by considering hexagonal honeycomb structures. Such structures initially show a linear-elastic deformation with slight bending of the cell walls when they are compressed in the transverse direction. When a critical stress value is reached, the cells collapse due to cell wall failure, which is manifested by a plateau in the stress–strain diagram with almost constant stress. A further increase of the stress leads to compressing the pores until almost no pores are left in the material^35^.

For axial loading of the wood, other failure mechanisms are considered. First, an elastic compression of the honeycomb segments takes place along the stress direction in which the cell walls swell out. When a critical failure stress is exceeded, failure occurs due to buckling of the cell walls. The buckling can be either plastic or brittle depending on the type of wood. The required buckling stress in the axial case, however, is significantly higher than in the transversal direction, since the cell walls are at a certain angle to each other (120° in hexagonal honeycombs), whereby they stiffen each other, and an increase in the geometrical moment of inertia occurs^35^.

### Fire resistance

**Figure 6:**
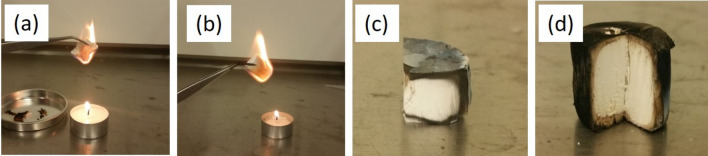
Results of the fire behavior tests of the freeze casts. (**a**) Sample 10-0 burns off completely. (**b**) The sample 10–0.5 MFC also burns off completely. (**c**) Sample 10-10-SiO_2_ after about one minute flame exposure. (**d**) Sample 10–5 NC after one minute of flame exposure.

When speaking about building materials, attention should be paid to the safety of all occupants. For this reason, it is necessary that the building material is incombustible, to prevent the risk of a fire. UL94, CONE and LOI are well known methods to check flammability and/or weight loss after heating. Due to the presence of PVA in our samples, we thought of using the method LOI, but with this method we would have obtained information about the decomposition temperature of the PVA rather than direct information about the "flammability" of our of PVA freeze casts and composites. Although being aware of these methods, we decide to perform simple tests over an open flame (Fig. 6). For comparison, commercially available PU foam (tecta-PUR, Karl Bachl Kunststoffverarbeitung GmbH and Co. KG), XPS (JACKODUR, Jackon Insulation GmbH) and our freeze casts were tested.

The pure PVA samples and the MFC/PVA composite immediately caught fire at contact with the flame and then burned completely. The results for the freeze casts with SiO_2_ and nano-clay were significantly better. They fired less quickly, hardly glowing, and once the surface was completely burnt, their interior remained intact. Upon renewed flame exposure, the pieces did not burst into flame again. The PU foam showed a very fast flammability and strong smoking. However, this foam did not completely burn off, presumably due to the use of flame retardants in the commercial product. The XPS caught immediately fire and burned completely with burning drops.

## Conclusions

We were able to produce highly porous PVA foams as well as composites with SiO_2_, MFC and NC by the ice-template-process. The total pore volume of the materials decreases with increasing solid content (PVA + additives). The diameter of the pore channels varies between about 15 to 40 microns.

The thermal conductivities of the freeze casts are well within the range of common insulating materials. Particularly in transversal direction very low values arise depending on the composition and solids content. They range from 0.030 to 0.075 W m^−1^ K^−1^. The thermal conductivity increases when the amount of solid content increases. The compressive strength of the freeze casts strongly depends on the load direction. In the case of axial loading, values are significantly higher. The highest compressive strengths are achieved with high PVA content and the addition of SiO_2_.

However, the freeze casts with the highest compressive strength are not the ones with the lowest thermal conductivity. Thus, a compromise between insulation effect and strength can be chosen combining the two properties as well as possible. The freeze casts with MFC seem to be the most suitable. Even small amounts of fibers provide a significant increase in strength, while the thermal conductivity is similar to that of pure PVA freeze casts. Optionally, the combination of fibers and SiO_2_ particles can lead to further property improvement. The addition of clay minerals, however, shows no advantages over the other additives. Concerning the pores inside the freeze casts: the pore size can be adjusted to a certain extent by controlling the freezing rate of the solvent. This can be either done by adjusting the temperature of the bottom plate^36^ or by slowly submerging the sample container into the cooling fluid^37^. The main disadvantage of the freeze-casting technique is the lack of scalability to larger sizes. While an increase of the sample size in lateral direction (perpendicular to the freezing direction) could be easily realized by increasing the size of the cooling surface, an increase along the pore channels is limited since the solidifying solvent is insulating the cooling surface from the solution. This leads to a decrease in freezing rates until the directional growth is interrupted (Supplementary Information).

## Supplementary Information

Supplementary Figures.

## Data Availability

The datasets used and/or analysed during the current study are available from the corresponding author on reasonable request.
