# Supplementary material for: Poly(vinyl alcohol) freeze casts with nano-additives as potential thermal insulators
Source: Sci Rep. 2023 Jan 19;13:1020. doi: 10.1038/s41598-022-27324-2 (PMC9852270; doi:10.1038/s41598-022-27324-2)
Supplement: Supplementary file 1 — Supplementary Figures. [file 41598_2022_27324_MOESM1_ESM.docx]

**Supplementary information**


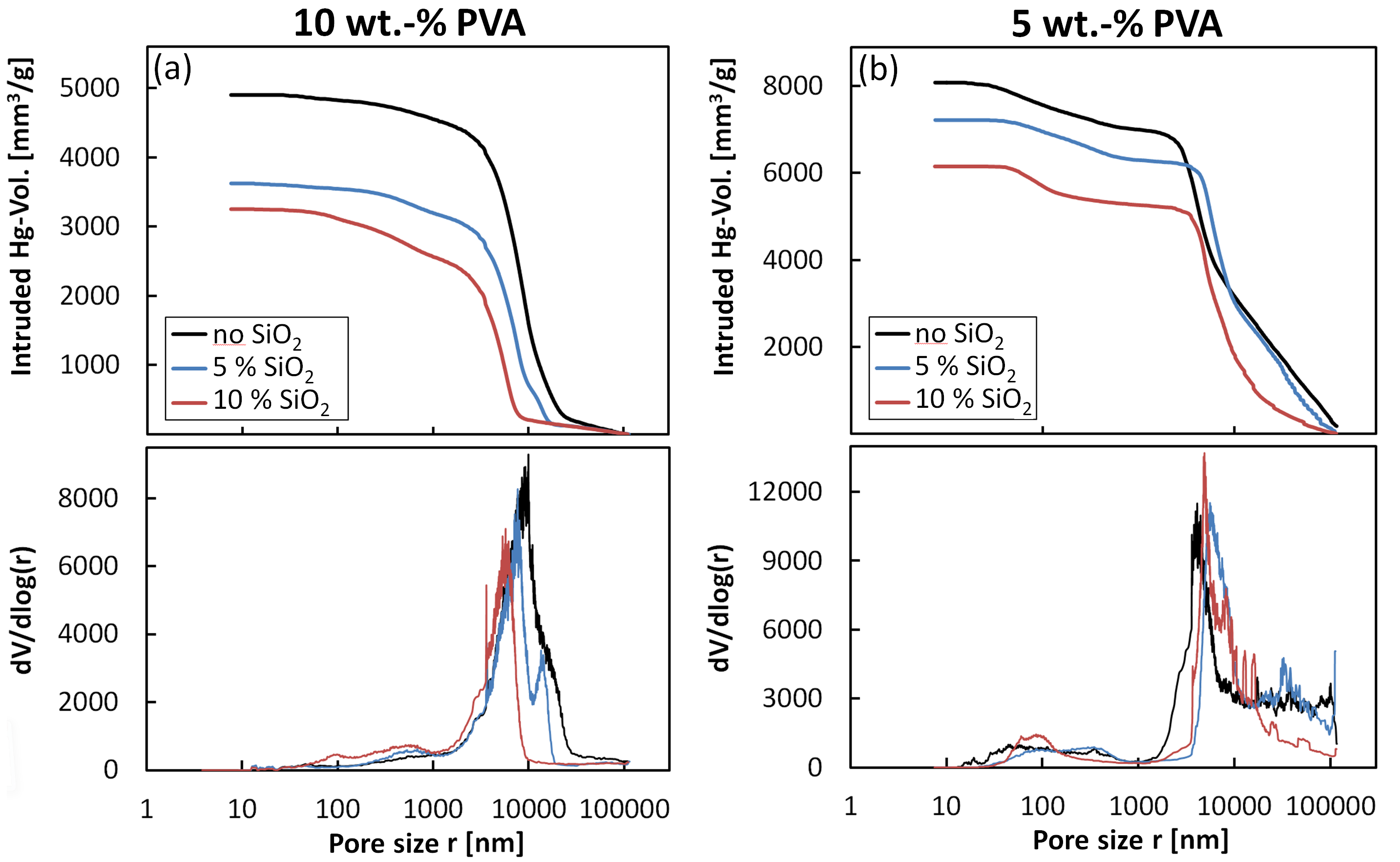


**Figure S1:** Mercury porosimetry results of freeze-cast PVA samples with different amounts of SiO_2_ nanoparticles. (a) 10 wt.-% PVA. (b) 5 wt.-% PVA.


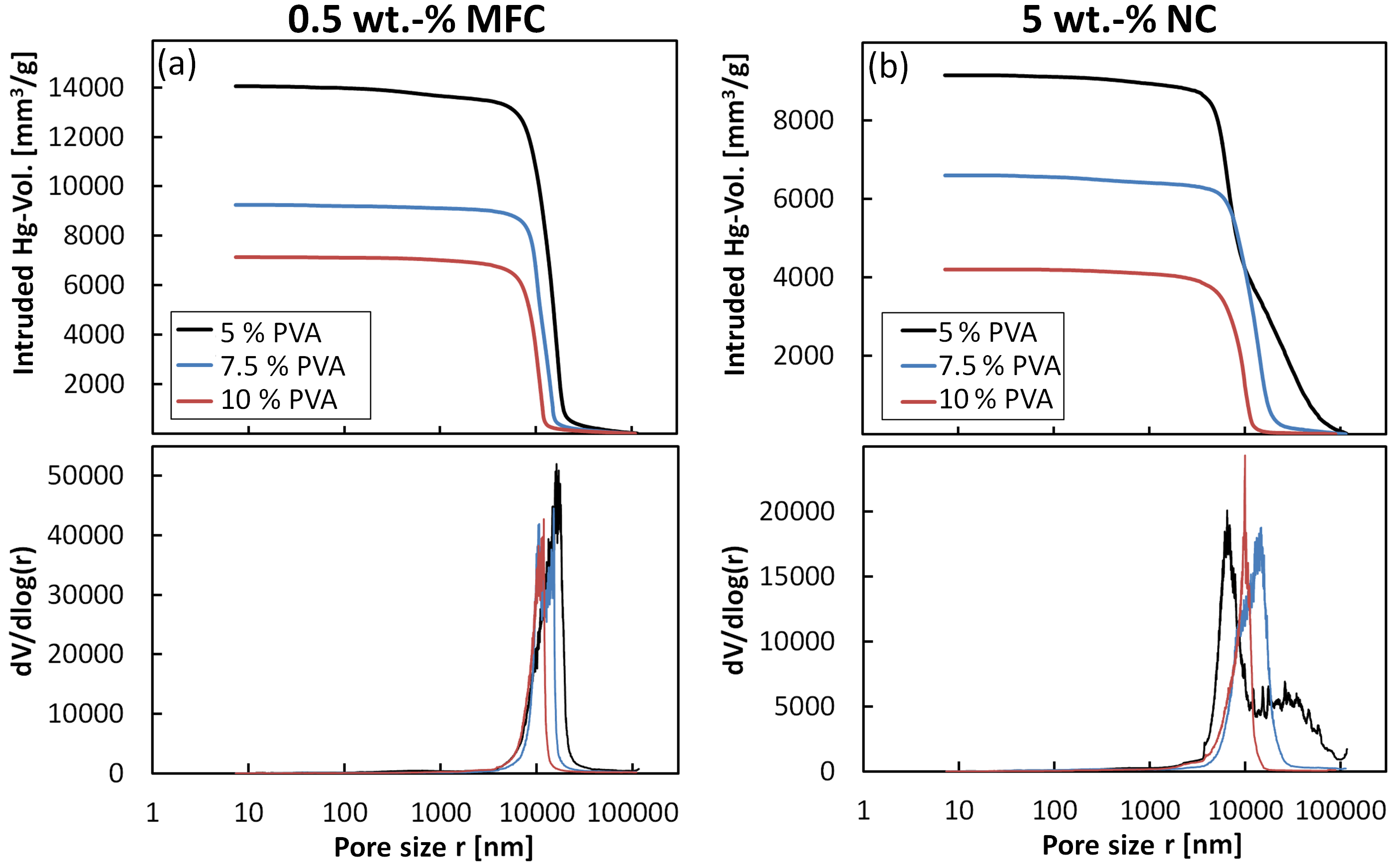


**Figure S2:** Mercury porosimetry results of freeze-cast samples with different amounts of PVA and (a) microfibrillated cellulose (MFC) fibers and (b) nano-clay (NC).
